# 166例双表达弥漫大B细胞淋巴瘤临床特征与预后分析

**DOI:** 10.3760/cma.j.issn.0253-2727.2022.09.010

**Published:** 2022-09

**Authors:** 书翰 唐, 磊 田, 伟 赵, 晶 王, 晓燕 克

**Affiliations:** 北京大学第三医院血液内科，北京 100083 Department of Hematology, Peking University Third Hospital, Beijing 100083, China

**Keywords:** 淋巴瘤，大B细胞，弥漫性, 临床特征, 预后, 基因，BCL2, 双表达淋巴瘤, Lymphoma, large B-cell, diffuse, Clinical features, Prognosis, Gene, BCL2, Double expression lymphoma

## Abstract

**目的:**

探讨双表达弥漫大B细胞淋巴瘤（DLBCL）的临床特征与预后。

**方法:**

回顾性分析2016年1月至2020年12月北京大学第三医院收治的166例双表达DLBCL患者的临床资料，分析患者的临床特征、生存和预后因素。

**结果:**

共收集DLBCL患者410例，其中双表达淋巴瘤（DEL）166例（40.5％）。男82例，女84例，中位诊断年龄63.5（21～95）岁。110例（66.3％）患者初诊年龄≥60岁，106例（106/163，65.0％）患者诊断时LDH升高，74例（74/160，46.2％）诊断时β_2_-微球蛋白（β_2_-MG）≥3 mg/L，107例（107/163，65.6％）结外受累数目≥2个，65例（65/166，39.2％）有B症状，131例（131/165，79.4％）初诊时分期为Ⅲ、Ⅳ期，41例（41/161，25.5％）初诊时国际预后指数（IPI）评分0～2分，38例（38/161，23.6％）初诊时IPI评分3分，82例（82/161，50.9％）初诊IPI评分4～5分。DEL患者中9例（9/56，16.1％）具有MYD88和CD79B突变。单因素分析显示，年龄≥60岁（*P*＝0.004）、β_2_-MG水平升高（*P*＝0.002）、IPI评分高（*P*＝0.003）与较差的总生存（OS）相关，β_2_-MG水平升高（*P*＝0.031）、LDH水平升高（*P*＝0.017）、分期Ⅲ～Ⅳ期（*P*＝0.001）、IPI评分高（*P*＝0.013）、免疫组化p53突变型（*P*＝0.049）和PIM1突变（*P*＝0.039）与较差的无进展生存（PFS）相关。多因素分析显示，IPI评分4～5分（*HR*＝2.622，95％*CI* 1.398～4.917，*P*＝0.003）是影响DEL患者OS的独立危险因素。生存分析显示，DEL与非DEL患者的PFS率差异有统计学意义（65.6％对75.1％，*P*＝0.002），而OS率的差异无统计学意义（81.8％对83.6％，*P*＝0.226）。在DEL患者中，REPOCH方案的总有效率高于RCHOP或RCHOP样方案（81.5％对63.4％，*P*＝0.004）。

**结论:**

DEL是一组侵袭性较强的淋巴瘤，具有较差的PFS，采用REPOCH方案化疗可能会改善患者的整体预后。

## Abstract (en)

**Objective:**

To investigate the clinical features and prognosis of MYC/BCL2 double-expression diffuse large B-cell lymphoma（DEL）.

**Methods:**

The clinical data, including clinical characteristics, survival, and prognostic factors, of 166 patients with DEL treated at Peking University Third Hospital from January 2016 to December 2020 were retrospectively analyzed.

**Results:**

A total of 410 patients with diffuse large B-cell lymphoma were collected, including 166 cases（40.5％）of DEL. There were 82 males and 84 females with a median age of 63.5（21–95）years at diagnosis. A total of 110 patients（66.3％）were aged over 60 years at initial diagnosis, 106 patients（106/163, 65.0％）had elevated lactate dehydrogenase（LDH）at diagnosis, 74 patients（74/160, 46.2％）had β_2_ microglobulin level over 3 mg/L at diagnosis, and 107 patients（107/163, 65.6％）had≥2 extranodal involvement. Sixty-five patients（65/166, 39.2％）had B symptoms, 131 patients（131/165, 79.4％）had stage Ⅲ and Ⅳ disease at initial diagnosis, 41 patients（41/161, 25.5％）had an International Prognostic Index（IPI）score of 0–2 at initial diagnosis, and 38 patients（38/161, 23.6％）had an IPI score of 3 at initial diagnosis. Eighty-two patients（82/161, 50.9％）had an IPI score of 4–5 at initial diagnosis. Nine（9/56, 16.1％）patients with DEL had MYD88 and CD79B mutations. Univariate analysis showed that age over 60 years（*P*＝0.004）, increased β_2_ microglobulin level（*P*＝0.002）, and high IPI score（*P*＝0.003）were associated with poor overall survival（OS）. Increased β_2_ microglobulin level（*P*＝0.031）, LDH level（*P*＝0.017）, stage Ⅲ–Ⅳ（*P*＝0.001）, high IPI score（*P*＝0.013）, immunohistochemical p53 mutation（*P*＝0.049）, and PIM1 mutation（*P*＝0.039）were associated with poor progression-free survival（PFS）. Multivariate analysis showed that IPI score of 4–5 was an independent risk factor for the prognosis of DEL（*HR*＝2.622, 95％ *CI* 1.398–4.917, *P*＝0.003）. Survival analysis showed that there was a significant difference in the PFS between patients with DEL and those without DEL（65.6％ *vs* 75.1％, *P*＝0.002）. However, there was no significant difference in the OS（81.8％ *vs* 83.6％, *P*＝0.226）. In patients with DEL, the overall response rate of R-EPOCH regimen was higher than that of RCHOP or RCHOP-like regimen（81.5％ *vs* 63.4％, *P*＝0.004）.

**Conclusion:**

DEL is a group of aggressive lymphomas with relatively poor PFS. The R-EPOCH regimen may improve the overall prognosis of patients.

弥漫大B细胞淋巴瘤（DLBCL）是最常见的侵袭性非霍奇金淋巴瘤（NHL），临床上具有明显的异质性，根据基因表达谱和免疫组化方法对DLBCL进行细胞起源（COO）分型，可分为生发中心来源（GCB）和非GCB（non-GCB）两种亚型，该分型方法对于预后的价值已得到广泛接受和认可，但仍有部分患者的预后具有差异性。在2016版世界卫生组织（WHO）关于造血和淋巴系统肿瘤的分类中将伴有c-myc和bcl-2和（或）bcl-6基因重排正式定义为高级别B细胞淋巴瘤（HGBL）[1]，具有c-myc和bcl-2或bcl-6易位的B细胞淋巴瘤被称为双打击淋巴瘤（DHL），具有三个基因易位的B细胞淋巴瘤被称为三打击淋巴瘤（THL）。免疫组化染色提示Myc和Bcl-2蛋白高表达，而FISH分析未显示基因重排，被称为双表达淋巴瘤（DEL）[2]–[5]。

目前研究认为DEL的预后优于DHL，但与普通DLBCL相比生存率仍较差[6]。由于其诊断标准（Myc和Bcl-2阳性阈值）及预后仍存在争议，且NCCN指南中并未提出统一有效的治疗方案，因此目前并未将其列为独立的诊断类型，对于此类患者是否需要调整治疗策略也无定论。基于此，本研究回顾性分析了北京大学第三医院血液科收治的166例DEL患者，对患者的临床资料进行分析，并进一步探讨DEL患者的诊断和治疗策略。

## 病例与方法

1. 病例：本研究回顾性收集了2016年1月至2020年12月北京大学第三医院就诊的410例初治DLBCL患者的临床资料，所有患者均根据2008版WHO造血与淋巴系统肿瘤分类诊断标准经过病理组织活检和（或）免疫组织化学染色进行诊断。通过免疫组织化学染色检测肿瘤细胞中Myc、Bcl-2的表达，Myc≥40％且Bcl-2≥50％定义为DEL，通过荧光原位杂交（FISH）检测有无c-myc、bcl-2和（或）bcl-6基因重排。410例初治DLBCL患者中DEL患者166例（40.5％），其中114例完善了FISH相关检查，10例（10/114，8.8％）患者为DHL。

2. 基因突变检测：在DEL患者中，56例患者进行了高通量测序基因突变检测，检测的基因包括B2M、BCL2、BCL6、CARD11、CD79A、CD79B、KMT2D、MYC、MYD88、PIM1、STAT6、TP53等55个基因。

3. 治疗方案：166例DEL患者中82例接受了RCHOP方案（利妥昔单抗+环磷酰胺+阿霉素+长春新碱+泼尼松）或RCHOP样方案化疗，65例接受REPOCH方案（利妥昔单抗+依托泊苷+泼尼松+长春新碱+环磷酰胺+阿霉素）化疗，11例接受了COPADM方案（环磷酰胺+长春新碱+大剂量甲氨蝶呤+阿霉素）、R-GDP方案（利妥昔单抗+吉西他滨+顺铂+地塞米松）、TEDDi-R方案（替莫唑胺+依托泊苷+脂质体阿霉素+地塞米松+利妥昔单抗）、R-hyper CVAD（环磷酰胺+长春新碱+阿霉素+地塞米松+利妥昔单抗）等其他化疗方案的治疗，8例未接受系统治疗。在接受系统治疗的158例患者中，20例患者接受了自体造血干细胞移植（auto-HSCT）。本研究中，化疗方案未进行剂量调整，高龄患者减量1/4，如治疗期间出现粒细胞缺乏伴发热会继续减量。

4. 随访：采用门诊或电话联系的方式随访，随访截止时间为2021年4月。无进展生存（PFS）时间定义为自患者诊断至首次出现疾病进展、死亡或随访截止的时间。总生存（OS）时间定义为自患者诊断至因任何原因死亡或随访截止的时间。

5．统计学处理：采用SPSS 20.0软件进行统计学分析，*P*<0.05为差异有统计学意义。计数资料采用例数（百分比）形式描述，计量资料采用中位数（范围）描述。预后影响因素的单因素分析采用Kaplan-Meier法和Log-rank检验，并绘制生存曲线，采用Cox比例风险回归模型进行多因素分析。

## 结果

1. 临床特征：本研究共纳入166例初诊DEL，其中男82例，女84例，中位诊断年龄63.5（21～95）岁，110例（66.3％）患者初诊年龄≥60岁，106例（106/163，65.0％）患者诊断时LDH升高，74例（74/160，46.2％）诊断时β_2_-微球蛋白（β_2_-MG）≥3 mg/L，107例（107/163，65.6％）结外受累数目≥2个，65例（39.2％）有B症状，131例（131/165，79.4％）初诊时分期为Ⅲ、Ⅳ期，41例（41/161，25.5％）初诊时国际预后指数（IPI）评分0～2分，38例（38/161，23.6％）初诊时IPI评分3分，82例（82/161，50.9％）初诊IPI评分4～5分。DEL患者与非DEL患者年龄（*P*＝0.042）、分期（*P*＝0.034）、IPI评分（*P*＝0.002）和β_2_-MG水平（*P*＝0.002）的差异有统计学意义（表1）。

**表1:** 双表达淋巴瘤（DEL）患者与非DEL患者临床特征比较［例（％）］

特征	DEL（166例）	非DEL（244例）	*χ*^2^值	*P*值
年龄			4.341	0.042
<60岁	56（33.7）	118（48.4）		
≥60岁	110（66.3）	126（51.6）		
性别			0.080	0.777
男	82（49.4）	124（50.8）		
女	84（59.6）	120（49.2）		
B症状			2.520	0.112
有	65（39.2）	77（31.6）		
无	101（60.8）	167（68.4）		
Ki-67			28.915	<0.001
≥80％	125（75.3）	120（58.3）		
<80％	41（24.7）	86（41.7）		
细胞来源			8.517	0.004
非GCB	135（81.3）	140（68.0）		
GCB	31（18.7）	66（32.0）		
分期			4.482	0.034
Ⅰ～Ⅱ	34（20.6）	73（30.2）		
Ⅲ～Ⅳ	131（79.4）	169（69.8）		
结外受累数目			0.622	0.430
0～1	56（34.4）	93（38.4）		
≥2	107（65.6）	149（61.6）		
IPI评分			12.186	0.002
0～2分	41（25.5）	98（40.5）		
3分	38（23.6）	59（24.4）		
4～5分	82（50.9）	85（35.1）		
LDH（U/L）			3.809	0.051
>245	106（65.0）	135（55.3）		
≤245	57（35.0）	109（44.7）		
β_2_-MG（mg/L）			14.529	0.002
1～3	86（53.8）	155（65.1）		
≥3	74（46.2）	83（34.9）		

注：GCB：生发中心来源；IPI：国际预后指数；LDH：乳酸脱氢酶；β_2_-MG：β_2_-微球蛋白

2. 免疫组化检测：166例DEL患者初诊时的免疫组化结果显示，135例（81.3％）为non-GCB亚型，31例（18.7％）为GCB亚型；143例（86.1％）Ki-67≥70％，125例（75.3％）Ki-67≥80％，61例（36.7％）Ki-67≥90％；54例（32.5％）Myc≥50％，21例（12.7％）Myc≥70％；69例（41.6％）Bcl-2≥70％，32例（19.3％）Bcl-2≥90％；36例（36/86，41.9％）患者p53为突变型。

3. 基因突变分析：166例DEL患者中56例进行了二代测序基因突变检测，56例患者均存在基因突变，其中IGLL5、PIM1、CD79B、MYD88、KMT2D、BTG1、TP53、PRDM1、DTX、BTG2、PCLO、TMSB4X、BCL2、CREBBP和CCND3的突变频率大于10％，CD79B和MYD88双突变患者9例（9/56，16.1％），TP53突变患者10例（10/56，17.8％）（图1）。

**图1:**
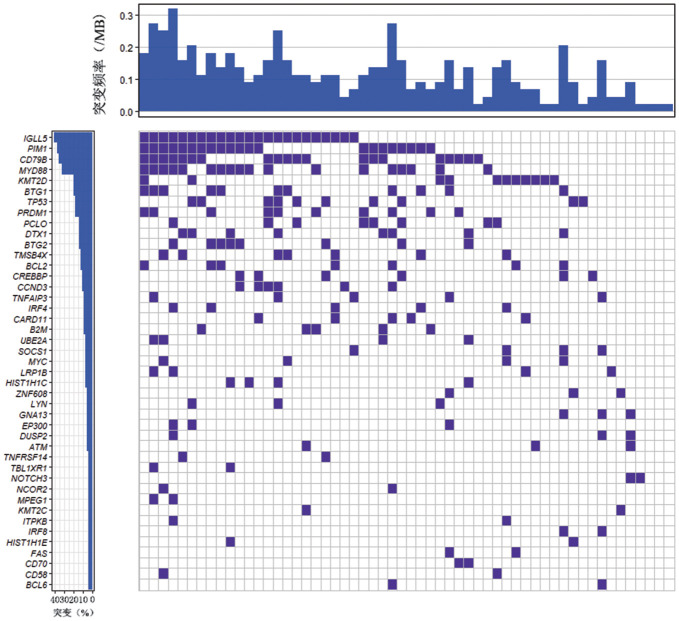
56例双表达淋巴瘤患者二代测序基因突变检测结果

相关性分析显示，IGLL5突变与高分期和B症状相关，PIM1突变与年龄相关。其余基因突变与性别、年龄、结外累及、骨髓受累、分期、有无B症状、LDH水平、β_2_-MG水平、IPI评分均无关。

4. 治疗：166例DEL患者中158例进行了化疗，其中82例接受RCHOP或RCHOP样方案化疗，65例接受REPOCH样方案化疗，11例接受其他方案化疗。接受6个周期化疗后进行评估，所有DEL患者初始治疗后总有效率（ORR）为68.3％（108/158），完全缓解（CR）率为50.6％（80/158），部分缓解（PR）率为17.7％（28/158）。其中，RCHOP或RCHOP样方案化疗的ORR为63.4％（52/82），CR率为42.7％（35/82），PR率为20.7％（17/82）。REPOCH方案化疗的ORR为81.5％（53/65），CR率为64.6％（42/65），PR率为16.9％（11/65）。对于DEL患者，REPOCH方案组的ORR优于RCHOP或RCHOP样方案组，差异有统计学意义（*P*＝0.004）。

在接受化疗的158例DEL患者中，120例完成了6个疗程的化疗，CR率为56.7％（68/120），PR率为20％（24/120），ORR为76.7％（92/120）；38例未完成6个疗程的化疗，CR率为31.6％（12/38），PR率为7.9％（3/38），ORR为39.5％（15/38）。75岁及以上的DEL患者32例，14例未完成化疗，16例完成6个疗程的化疗，2例未接受治疗。完成化疗的16例患者的CR率为31.3％（5/16），PR率为37.5％（6/16），ORR为68.8％（11/16）。

244例非DEL患者中232例进行了化疗，12例患者未接受治疗。接受化疗的232例患者中168例接受RCHOP或RCHOP样方案化疗，55例接受REPOCH样方案化疗，9例接受其他方案化疗。接受6个周期化疗后进行评估，所有非DEL患者初始治疗后ORR为71.1％（165/232），CR率为57.3％（133/232），PR率为13.8％（32/232）。其中，接受RCHOP或RCHOP样方案化疗患者的ORR为72％（121/168），CR率为57.1％（96/168），PR率为14.9％（25/168）；接受REPOCH方案化疗患者的ORR为70.9％（39/55），CR率为60.0％（33/55），PR率10.9％（6/55）。对于非DEL患者，REPOCH方案组与RCHOP或RCHOP样方案组疗效的差异无统计学意义（*P*＝0.744）。

DEL组分析结果显示，接受RCHOP样方案治疗的82例患者中，non-GCB亚型70例，复发率为41.4％（29/70），死亡率为18.6％（13/70）；GCB型12例，复发率为16.7％（2/12），死亡率为8.3％（1/12）。接受REPOCH方案治疗的65例患者中，non-GCB亚型52例，复发率为36.5％（19/52），死亡率为13.5％（7/52）；GCB亚型13例，复发率为30.8％（4/13），死亡率为0。

166例DEL患者中，158例接受系统治疗，其中20例患者接受了auto-HSCT，138例患者未进行auto-HSCT，移植组和非移植组的3年OS率分别为90％和85.5％（*P*＝0.532）；1年复发率分别为5％和24.6％（*P*＝0.049）。

20例接受auto-HSCT的患者中男12例，女8例，5例（25％）患者初诊时年龄≥60岁，15例（75％）为non-GCB亚型，18例（90％）患者Ki-67≥70％，17例（85％）Ki-67≥80％，11例（55％）Ki-67≥90％；5例（25％）患者Myc≥50％，4例（20％）Myc≥70％；8例（40％）患者Bcl-2≥70％，3例（15％）Bcl-2≥90％。13例（65％）患者p53为突变型，11例（55％）诊断时LDH升高，7例（35％）诊断时β_2_-MG≥3 mg/L，14例（70％）结外受累数目≥2，7例（35％）有B症状，17例（85％）初诊时Ann Arbor分期Ⅲ～Ⅳ期，10例（50％）初诊时IPI评分为0～2分，5例（25％）初诊IPI评分3分，5例（25％）初诊IPI评分为4～5分，3例（15％）有骨髓受累，19例（95％）移植时已达到CR。

5．预后因素及生存：410例DLBCL患者中接受系统治疗的可随访患者367例，中位随访时间为18（0～75）个月，1年、3年、5年OS率分别为89.6％、85.9％、82.8％，1年、3年、5年PFS率分别为81.2％、72.5％、71.1％。其中154例DEL患者1年、3年、5年OS率分别为87.7％、83.8％、81.8％，1年、3年、5年PFS率分别为77.9％、68.2％、65.6％。213例非DEL患者1年、3年、5年OS率分别为91.1％、85.9％、83.6％，1年、3年、5年PFS率分别为83.6％、75.6％，75.1％。DEL与非DEL患者PFS率的差异有统计学意义（*P*＝0.002），OS率的差异无统计学意义（*P*＝0.226）（图2）。

**图2:**
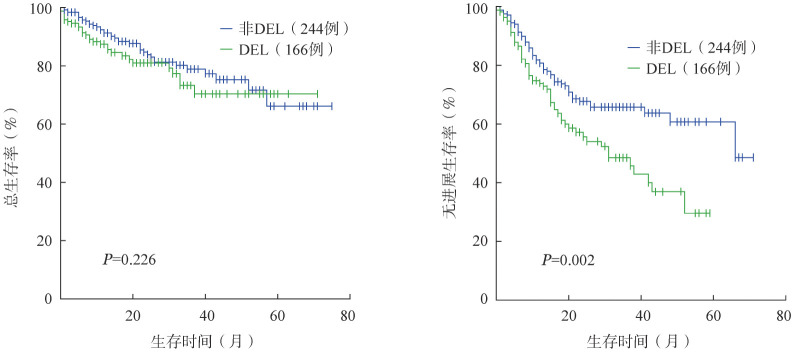
双表达淋巴瘤（DEL）与非DEL患者的总生存（A）和无进展生存（B）曲线

CD79B和MYD88双突变的9例患者1年、3年OS率均为100％，1年、3年PFS率分别为88.9％和66.7％。进一步生存分析显示CD79B和MYD88双突变未导致预后不良。

本研究有81例DEL患者接受了中枢神经系统预防，共有7例中枢神经系统受累患者，其中3例（3.7％）为中枢神经系统原发DEL，4例（4.9％）为中枢神经系统继发DEL。81例DEL患者1年、3年、5年OS率分别为90.4％、76.5％、76.5％，1年、3年、5年PFS率分别为63.0％、42.0％、37.0％。

单因素分析显示，年龄≥60岁（*P*＝0.004）、β_2_-MG水平升高（*P*＝0.002）、IPI评分高（*P*＝0.003）与较差的OS相关，β_2_-MG水平升高（*P*＝0.031）、LDH水平升高（*P*＝0.017）、分期Ⅲ～Ⅳ期（*P*＝0.001）、IPI评分高（*P*＝0.013）、免疫组化p53突变型（*P*＝0.049）和PIM1突变（*P*＝0.039）与较差的PFS相关。多因素分析显示，IPI评分4～5分（*HR*＝2.622，95％*CI* 1.398～4.917，*P*＝0.003）是影响DEL患者OS的独立危险因素。本研究中Ki-67以70％、80％、90％作为界值，Myc蛋白以50％、60％、70％作为界值，各组预后的差异均无统计学意义（*P*值均>0.05）。

## 讨论

本研究中DEL的发病率为40.5％，Hwang等[7]报道的一项Meta分析中提到，DEL的发病率为6％～50％，偏倚调整后的发病率为31％（95％*CI* 27％～36％）。本研究中DEL发病率偏高，我们收治的患者中部分是外院化疗后患者，可能存在选择偏倚。

多数研究表明，DEL患者预后较差，且更容易出现在高龄患者中，存在分期晚、结外受累及IPI评分高危等不良因素[8]–[9]。本研究中，与非DEL患者的基线特征比较，DEL具有高龄（>60岁）、疾病分期晚（Ⅲ～Ⅳ期），β_2_-MG水平高、IPI评分高，Ki-67高表达特点，与预后不良具有相关性，与既往文献报道基本相同[10]。本研究中DEL以non-GCB亚型为主，占81.2％，而GCB亚型仅占18.8％，与Ting等[11]的报道（两种亚型分别占74.2％和25.8％）基本相同。而Akyurek等[12]报道，DEL中两种亚型的比例分别为49.0％与51.0％。亚组分析显示，对于DEL患者，无论是R-CHOP样还是R-EPOCH样方案治疗，non-GCB亚型的复发率和死亡率均较GCB亚型高。

目前WHO建议以c-Myc≥40％、Bcl-2≥50％作为DEL诊断阈值，本研究中采用该定义。研究结果显示DEL组患者5年PFS率低于非DEL组患者（65.6％对75.1％，*P*＝0.002），5年OS率略低于非DEL组（81.8％对83.6％，*P*＝0.226）。目前不同研究对于c-Myc、Bcl-2的最佳阳性阈值存在争议，Johnson等[2]以c-Myc>40％和Bcl-2>50％作为阈值，DEL组显示出较差的5年OS率（39％对70％，*P*<0.001）和5年PFS率（44％对66％，*P*＝0.002）。与此同时，也有部分国内外研究采用c-Myc≥40％、Bcl-2≥50％作为阈值，结果显示，DEL组患者的OS率和PFS率仅略低于非DEL组，差异无统计学意义[13]。Zaiem等[14]认为，DLBCL中Ki-67≥80％与预后不良相关，但本研究中，Ki-67≥80％与Ki-67<80％的患者相比，OS及PFS差异均无统计学意义，可能是由于本研究DEL患者的Ki-67水平普遍较高，75.3％的DEL患者Ki-67水平超过80％。

既往研究认为，TP53突变与淋巴瘤的形成有关，是DLBCL患者的独立不良预后因素，且TP53突变与p53过表达相关[15]–[16]。本研究中，p53突变型与较差的PFS相关（*P*＝0.049），但p53突变型未显示较差的OS，可能与复发后的积极治疗改善了这类患者的预后有关。

NCCN指南一线推荐RCHOP方案作为治疗DLBCL的方案，更高强度的REPOCH方案作为DHL的治疗方案，但DEL的一线方案仍未得出统一结论。Ting等[11]的研究显示，DEL在老年患者中更为普遍，接受RCHOP样方案治疗的DEL患者的中位OS时间较非DEL患者缩短［（17.7±4.4）个月对（29.8±1.9）个月，*P*＝0.080］。CALGB 50303研究显示，与RCHOP方案相比，REPOCH方案的毒性更大，不能改善PFS或OS，但研究也指出，高IPI评分及具有不良预后因素患者能否获益尚不明确[17]。M.D. Anderson癌症中心的一项回顾性研究评估了16例接受R-EPOCH方案治疗的DEL患者的临床结局，结果显示，1年PFS率为65％，1年OS率为86％[18]。Aggarwal等[19]回顾性评估了16例DEL患者，R-CHOP组的复发率显著高于R-EPOCH组（80％对18％，*P*＝0.042）。本研究显示，在6个周期化疗后进行疗效评估，一线REPOCH方案化疗的疗效明显优于一线R-CHOP样方案化疗（*P*＝0.004）。

此外，DEL患者是否需要进行auto-HSCT以及移植时机目前也尚无统一认识。Oki等[20]的研究显示，对于DHL患者，auto-HSCT或异基因造血干细胞移植（26例）未使无事件生存或OS获益。另一项研究将12例DEL患者随机分组，未行造血干细胞移植患者的2年PFS率为29％，行造血干细胞移植患者的2年PFS率为60％[21]。Kim等[22]的一项研究纳入67例接受auto-HSCT的DLBCL患者，DEL组与非DEL组5年OS率与5年PFS率的差异无统计学意义（*P*值分别为 0.429和0.614）。本研究中20例DEL患者接受了auto-HSCT，1年复发率为5％，1年OS率为100％，3年OS率为90％。20例患者中，1例为疾病进展后行补救治疗，其余19例均在6或8个周期获得CR后进行auto-HSCT，在移植后的1年内未复发，3年复发率为26.3％（5/19）。根据上述结果，DEL患者在初始治疗获得CR后行auto-HSCT是能够获益的。

综上所述，本研究显示，DEL是一组侵袭性较强的淋巴瘤，患者具有高龄、疾病分期晚、β_2_-MG水平高、高IPI评分特点，病理类型主要为non-GCB，Ki-67高表达，PFS较差。高IPI评分、免疫组化p53突变是DEL患者的预后不良因素。采用高强度的REPOCH方案化疗、一线进行auto-HSCT和CAR-T细胞治疗可能会改善DEL患者的整体预后。本研究为单中心回顾性研究，部分老年患者的用药剂量偏低、随访时间不够是本文的局限之处。
